# Dual growth mode of boron nitride nanotubes in high temperature pressure laser ablation

**DOI:** 10.1038/s41598-019-52247-w

**Published:** 2019-10-30

**Authors:** Jun Hee Kim, Hyunjin Cho, Thang Viet Pham, Jae Hun Hwang, Seokhoon Ahn, Se Gyu Jang, Hunsu Lee, Cheol Park, Cheol Sang Kim, Myung Jong Kim

**Affiliations:** 1 Functional Composite Materials Research Center, Korea Institute of Science and Technology, Wanju, 55324 Republic of Korea; 2 Composite Materials Applications Research Center, Korea Institute of Science and Technology, Wanju, 55324 Republic of Korea; 3 Department of Bionanosystem Engineering, Graduate School, Jeonbuk National University, Jeonju, 54896 Republic of Korea; 4 Division of Mechanical Design Engineering, Jeonbuk National University, Jeonju, 54896 Republic of Korea; 5 Advanced Materials and Processing Branch, NASA Langley Research Center, Hampton, Virginia 23681 USA; 6 Security and Disruptive Technologies Research Centre, National Research Council Canada, 1200 Montreal Road, Ottawa, Ontario K1A 0R6 Canada; 7 Division of Nano & Information Technology, KIST School, Korea University of Science and Technology, Seoul, 02792 Republic of Korea

**Keywords:** Synthesis and processing, Nanoscale materials, Nanoparticles

## Abstract

The morphological analysis of the end of boron nitride nanotubes (BNNTs) using high-resolution transmission electron microscopy (HR-TEM) can provide valuable insight into the growth mechanism in high temperature pressure (HTP) laser ablation where the best quality of BNNT materials can be obtained so far. Two growth modes of BNNT coexisting during the synthesis process have been proposed based on HR-TEM observation and length analysis. One is the root growth mode, in which boron nitride (BN) species formed via the surface interaction between surrounding N_2_ molecules and boron nanodroplets incorporate into the tubular structure. Another mode called open-end growth mode means the prolongation of tube growth from the exposed BN edge surrounding the surface of boron nanodroplets which is constructed by the heterogeneous nucleation of absorbed BN radicals from the gas plume. The statistical data, the proportions of end structures and the length of BNNTs, could be fitted to two growth modes, and the open-end growth mode is found to be especially effective in producing longer nanotubes with a higher growth rate. The scientific understanding of the growth mechanism is believed to provide the control for optimized production of BNNTs.

## Introduction

Boron nitride nanotubes (BNNTs) share some fundamental similarities in atomic structure with carbon nanotubes (CNTs) in which sp^2^ hybridized boron and nitrogen atoms replace the carbon and arrange themselves in a hexagonal network. Because of the polarity of the B-N bond caused by the difference in electronegativity between boron (2.04) and nitrogen (3.04)^1,2^, the charge distribution in BNNT is asymmetric where electrons charge is spatially confined to nitrogen atoms, resulting in insulating behavior with a wide bandgap (5~6 eV)^3,4^. In contrast to CNT, the electrical property of BNNT is independent of the diameter and chirality of the nanotube^3^. Besides having several characteristics similar to CNT, such as superb mechanical strength^5–8^ and high thermal conductivity^9,10^, BNNTs also possess many distinct and exceptional properties, for example, high neutron absorption^11^, piezoelectricity^11–13^, high temperature oxidation resistance^14^, and biocompatibility^15^.

For decades, CNTs have earned a high reputation as one of the most studied nanomaterials, thanks to advances in its fabrication methods^16–18^ that guarantee high quality and a bulk quantity of the CNTs needed for fundamental research. Developing an efficient synthesis route for BNNTs, on the other hand, is particularly difficult. To date, most BNNT synthesis techniques follow the same approaches that have been previously applied to produce CNT, such as arc discharge^19–22^, laser ablation^23–26^, ball milling^27–30^, and chemical vapor deposition^31–33^, but with efficiency far behind CNT synthesis. In recent years, the progress on BNNT synthesis has made revolutionary steps toward producing high-quality BNNTs coming from the development of high temperature pressure (HTP) laser ablation^34^ and an advanced thermal plasma process^35,36^. BNNTs acquired by these methods have high crystallinity, small diameter, and few walls, and are readily commercialized. It was previously reported that thermal plasma-based methods could induce an unprecedentedly high production rate, thus, showing promising potential for BNNT synthesis at an industrial scale. For instance, hydrogen-assisted induction thermal plasma presented by Kim *et al*. at NRC produced high-quality BNNTs at the rate of 20 g/h, without using a metal catalyst^35^. They also suggested a growth mechanism of BNNTs in this method involving the catalytic activity of hydrogen^37^. An extended-pressure inductively coupled plasma system that offers excellent flexibility in the use of various boron precursors, developed by Zettle’s group from UC Berkeley, can yield BNNTs at an even higher rate (35 g/h); however, the purity of the final products is slightly inferior, because a minority of other boron nitride byproducts (nanoribbon, nanococoon) are also attained^36^.

For HTP laser ablation, the synthesis process is carried out at an elevated nitrogen pressure that is two to 20 times higher than atmospheric pressure^34^. Besides high crystallinity, and having few walls, the produced BNNTs bear an exceptional aspect ratio (length to diameter ratio). The productivity in this method, however, is only at gram scale, significantly lower than that in other thermal plasma processes, presumably because of the nature of the laser ablation process, in which the reaction zone is spatially confined in a small volume of molten boron limited by the size of the laser beam. Nonetheless, the potential for improving production efficiency in HTP laser ablation is predictable, but any attempts to do so requires a comprehensive understanding of the growth mechanism of BNNT that has not yet been acheived. Smith *et al*. at the NASA Langley Research Center reported that the growth of BNNTs was mostly root-based when ascending in the gas plume; however, their report was fairly inconclusive^34^. Moreover, a simulation modeling of laser vaporization presented by Gnoffo *et al*. at NASA provided some important information about the growth process, although its validity is not scientifically confirmed yet, because of the lack of experimental evidence^38^.

Herein, we proposed two growth modes of BNNT coexisting in HTP laser ablation based on the morphology of the nanotube from the HR-TEM analysis. Important structural features at the end of nanotubes, such as encapsulated boron nanoparticles or a capped h-BN layer, are discussed comprehensively, and the details can offer some valuable suggestions on the initial stages of the nucleation and growth process of BNNTs. Also, we further analyzed the differences of end structures and evaluated the growth rate in each mode by measuring the length of nanotubes. We expect that the two growth models introduced in this study in combination with previous theoretical studies can be applied to develop an optimal route for BNNT synthesis in the laser ablation method.

## Experimental Section

### BNNT synthesis

**Figure 1:**
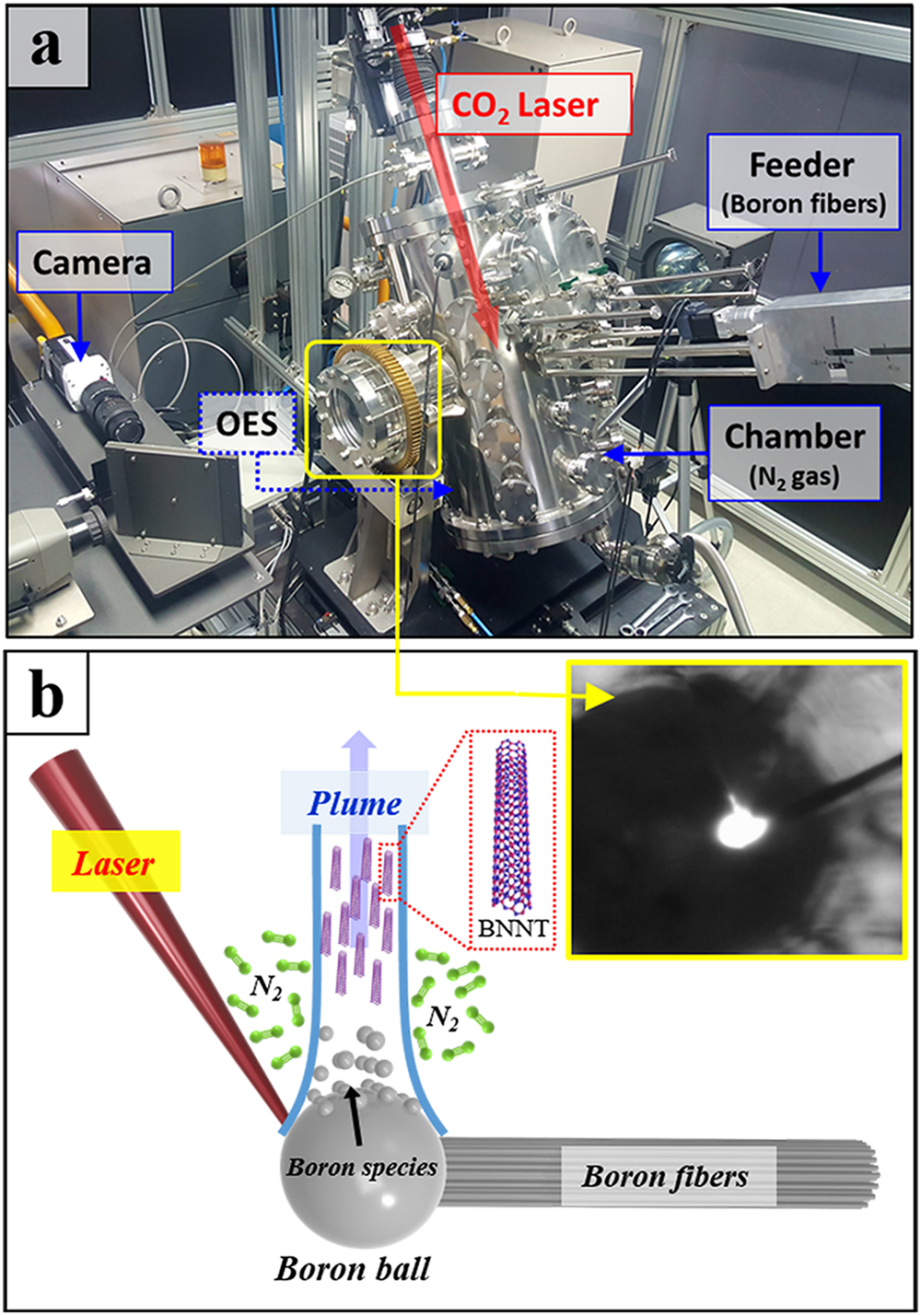
(**a**) Photo of HTP laser ablation system. (**b**) Illustration of BNNT synthesis in HTP laser ablation. Boron species evaporate on the surface of the molten boron ball under the continuous radiation of the CO_2_ laser and react with surrounding nitrogen molecules to form BNNTs (Inset shows the high-speed camera image of the molten boron ball).

The HTP laser ablation system (Laser oscillator, Laser C3000C, FANUC) for BNNT synthesis is shown in Fig. 1a. BNNTs were synthesized in a chamber specially designed, based on the reported HTP BNNT process^34,38^, to withstand high pressure and high temperature conditions. A bundle consisting of 70 boron fibers, each 102 μm in diameter, tied to a feeder, was placed in the center of the chamber filled with 14 bar of nitrogen (purity 99.999%). The number of boron fibers was optimized experimentally. If there were too many boron fibers, the bundle was not successfully melted, resulting in a decrease in yield. The bundle was irradiated continuously by a CO_2_ laser (wavelength 10.6 μm, original beam diameter 2 cm) focused down to 1 mm by a ZnSe lens throughout the synthesis process. We carried out *in situ* thermal analysis and close observation of the laser heating process by using a high-speed camera (Phantom Miro M110, Vision Research) and optical emission spectrometer (OES, Shamrock 500i, Andor).

The schematic of the laser ablation process in the BNNT synthesis is shown in Fig. 1b. The boron fibrous bundle was heated by 1000 W continuous CO_2_ laser, forming a spherical molten boron ball that had a temperature of 3845 K as measured by OES (Fig. S1). The temperature of the boron ball where it was directly irradiated by the laser could reach 5101 K, achieving the boiling point of boron at 14 bar, calculated from the Clausius-Clapeyron equation^38^, and causing the evaporation of the boron species. The gas plume rose vertically from the boron ball because of the buoyancy effect exerted by the surrounding N_2_. According to previous reports on BNNT synthesis in HTP laser ablation, the nucleation and growth of BNNTs were presumed to take place in the rising gas plume^34^. Cotton-like BNNT products were collected in collectors installed at different heights (1 cm and 13 cm) above the molten boron ball for qualitative comparison.

### Characterization

We conducted morphological and chemical analyses of raw BNNTs by using field emission scanning electron microscopy (FE-SEM, NOVA Nano SEM 450, FEI), high-resolution transmission electron microscopy (HR-TEM, Tecnai G2 F20, FEI), electron energy-loss spectroscopy (EELS), X-ray photoelectron spectroscopy (XPS, K-Alpha ESCA System, Thermo Scientific, Al Kα 1486.6 eV), and Raman spectroscopy (inVia, Renishaw, Ar laser λ = 514 nm). For length measurement, a BNNT solution was cast uniformly on a Si substrate by spray coating and subsequently dried at 60 °C for 24 hours. For preparation of the BNNT solution (as reported elsewhere^39^), the raw BNNT product was dispersed in DMAc solvent (0.25 mg/ml) by using bath sonication at low power for 30 minutes. We used FE-SEM to characterize separated BNNTs on the Si substrate. We measured the length of hundreds of BNNTs by using the Image J Program (NIH, USA) in FE-SEM images (Fig. S2). The proportions of the end structures were directly identified in the HR-TEM analysis.

## Results and Discussion

**Figure 2:**
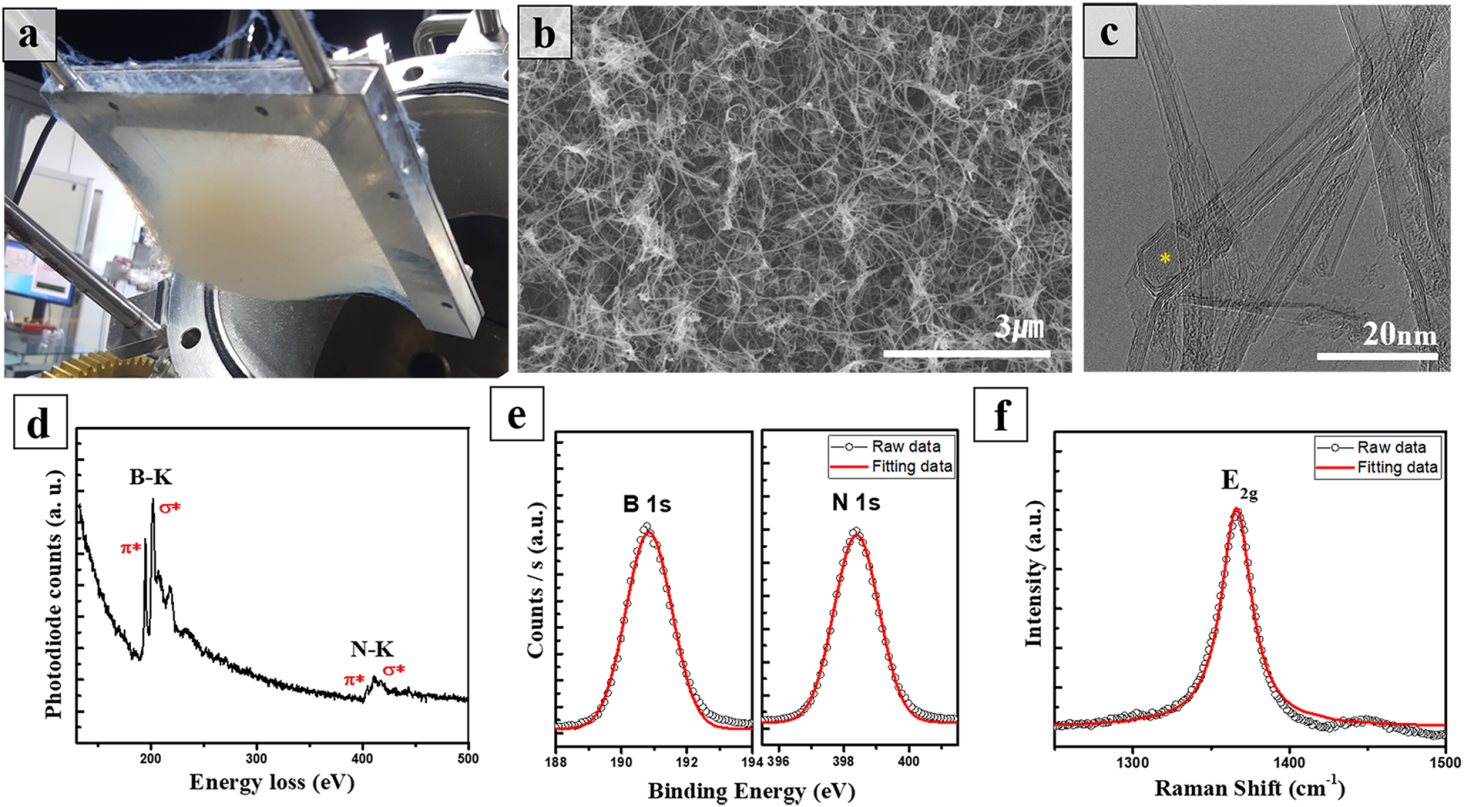
(**a**) Image of cotton-like BNNT web hanging on the collector after laser ablation, (**b**) FE-SEM image, and (**c**) HR-TEM image of raw BNNTs (yellow mark is a boron nanoparticle encapsulated at the end of BNNT). (**d**) Energy electron-loss spectrum of a BNNT shows K edges of boron and nitrogen with π* and σ* peaks attributed to sp^2^ hybridization. (**e**) High-resolution XPS spectra of raw BNNTs fitted with Gaussian curves display B 1 s and N 1 s core levels. (**f**) Raman spectrum of raw BNNTs fitted with Lorentzian curves indicates E_2g_ phonon mode of the hexagonal boron nitride atomic structure.

The cotton-like as-synthesized BNNTs were deposited on the collector as shown in Fig. 2a. The bright gray color of the sample represents a relatively high level of purity. The SEM image in Fig. 2b of the obtained BNNTs in HTP laser ablation shows the uniform distribution of entangled nanotubes with lengths up to more than 1 µm (Fig. S3a). Further details on the morphology of BNNTs were revealed in the HR-TEM analysis. As shown in Fig. 2c, we observed a variety of boron nitride nanostructures, such as BNNTs, h-BN, and boron nanoparticles in both collected samples. Most of the BNNTs, existing in either single tube or bundle form, had an average internal diameter of 4 nm, with the number of walls ranging from two to five, and high crystallinity (Fig. S3b). The stoichiometry of the nanotubes was determined in EELS measurement simultaneously with HR-TEM. The electron energy-loss spectrum in Fig. 2d displayed two prominent absorption peaks, located at 188 eV and 401 eV, corresponding to the K edges of boron and nitrogen, respectively^19^. A clear π* peak and σ* peak in both B-K and N-K edge regions are the signatures of sp^2^ hybridization^40^. The bonding states and chemical composition were further confirmed by XPS measurement (Fig. 2e). The atomic ratio of B to N ratio calculated from the high-resolution XPS peaks was close to 1:1, same as a result taken from EELS quantification analysis and in agreement with the stoichiometry ratio of B to N in a hexagonal boron nitride. The Raman spectrum of BNNTs in Fig. 2f exhibited a single peak at 1366.1 cm^−1^ corresponding to the E_2g_ phonon mode of a hexagonal boron nitride structure^41^.

**Figure 3:**
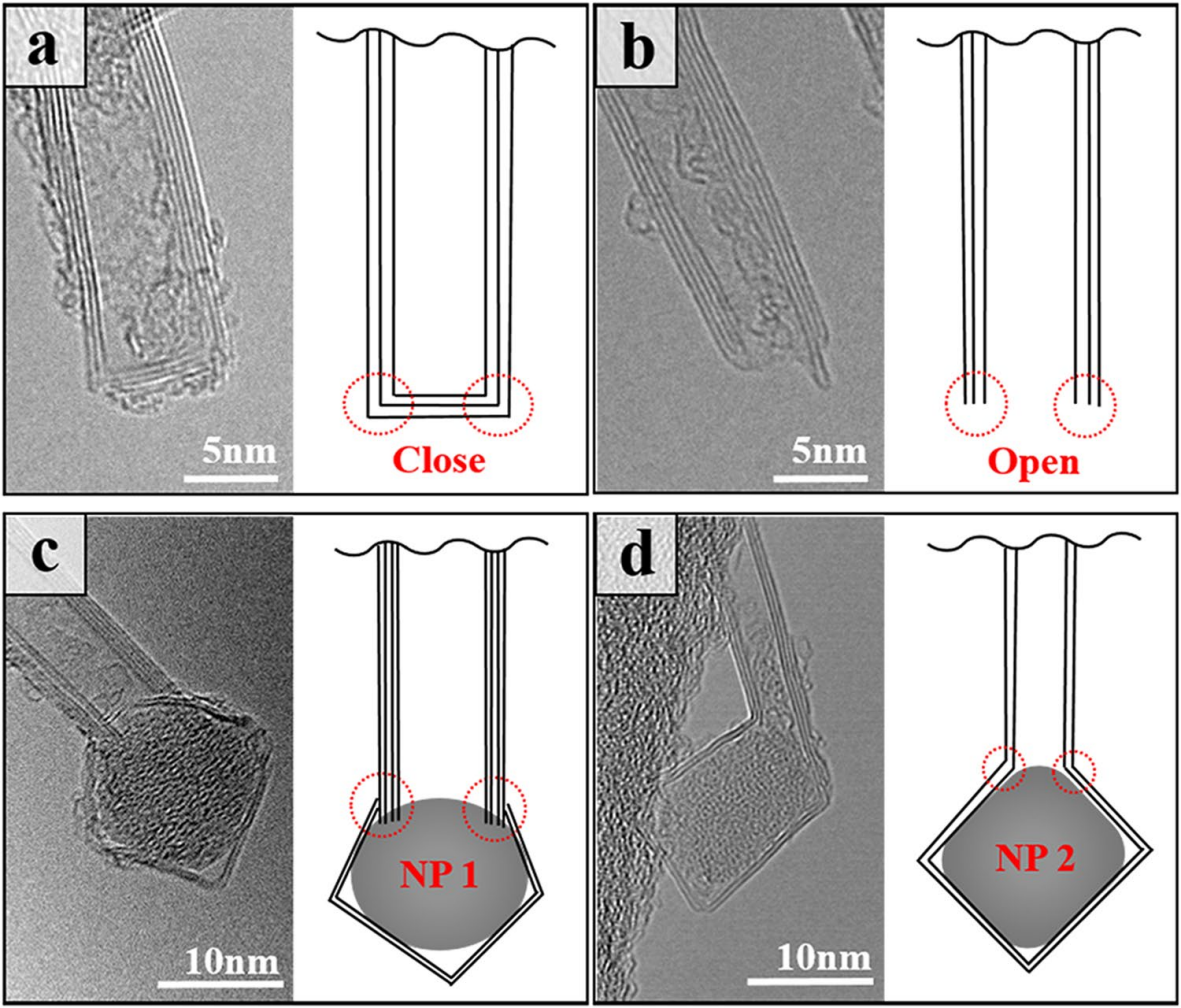
HR-TEM images of morphological structures at the end of BNNT: (**a**) flat end caused by the formation of even-numbered rings in the hexagonal network; (**b**) open end. (**c,d**) Two distinguishable nanoparticle-encapsulated end structures were observed in the HR-TEM analysis. In the NP1 end, a tubular structure grew directly from the surface of boron nanodroplets, whereas in the NP2 end, a BN nanotube was developed from polygonized h-BN layers wrapping around boron nanodroplets.

In most of the studies on one-dimensional tubular structures, the characteristics of a tube’s end provides valuable information about the growth process. From HR-TEM data as shown in Fig. 3, we recognized three characteristic features of the tip-ends of BNNTs: flat-closed, open, and nanoparticle-encapsulated (NP) end. We observed two distinguishable nanoparticle-encapsulated end structures in the HR-TEM analysis. In the NP1 end (Fig. 3c), tubular structure was grown directly from the surface of boron nanodroplets, whereas in the NP2 end (Fig. 3d), a BN nanotube was developed from polygonized h-BN layers wrapping around boron nanodroplets. These features at the ends are significant distinctions between CNTs and BNNTs. The hemispherical end in CNTs results from the formation of pentagonal and heptagonal defects in the atomic structure. These analogous defects in BNNTs would cause the energetically unfavorable B-B, and N-N bonds^42^ to destabilize the nanotube’s structure. Thus, to stop growing by capping, even-numbered rings, such as squares and octagons, are preferred, resulting in flat ends^43^. Furthermore, boron nanoparticles are commonly found at either end of the nanotubes, and they are still prevalent even in the catalytic synthesis. Metal catalysts, however, are rarely found at the end of BNNTs, and this is fundamentally opposed to the growth of CNTs, where growth is generally triggered from catalytic particles. Interestingly, BNNTs could start growing from the wrapped h-BN layer covered boron nanoparticles (Fig. 3d) or from the boron particle itself (Fig. 3c). This distinct feature on the origin of tube growth can give us some hints about a growth mechanism and will be thoroughly discussed in this study.

The formation of BNNTs is expected to be similar to that of CNTs, because BNNT has a similar hexagonal lattice tube structure with boron and nitrogen atoms by sp^2^ hybridization, and also a similar tube end structure. The generally accepted mechanism for the growth of CNTs in high temperature synthesis techniques, such as laser ablation and arc discharge, is a vapor–liquid–solid (VLS) model^44^. For CNTs, the model assumes that the first stage is the formation of a liquid metal carbide particle by the condensation of carbon and metal atoms from the vapor phase. Under the influence of the temperature and concentration gradient, solid-phase nanotubes begin to grow when metal particles become supersaturated with carbon species. After reaching the solubility limits, carbon species dissolved in the particles diffuse through the particles, begin to precipitate out, and form a graphitic cap on the surface (i.e., dissolution-precipitation mechanism). As carbon is continuously absorbed from surrounding carbon sources, the graphitic cap subsequently evolves into a cylindrical structure. This formation process is a well-known root growth mechanism. A similar scenario would happen in the growth of BNNTs. However, boron particles are more commonly found as the growth support of nanotube than are metal particles, and the dissolution-precipitation mechanism is no longer valid. The NP 1 end structures (Fig. 3c) strongly suggest that the formation of BNNT follows the VLS model.

The root-based growth mechanism of BNNT has also been previously reported^25,26,34,45–47^ in the arc discharge and laser ablation processes. In HTP laser ablation, the boron fibers are initially heated by the laser in 14 bar of nitrogen inside the chamber and melted down, forming a macroscopic boron ball. When reaching boiling temperature (5101 K), the boron ball starts evaporating, generating a vertical gas plume. Once the boron vapor in the gas plume condenses into molten boron nanodroplets as the temperature decreases, the root growth of BNNTs is initiated and proceeds as follows.

**Figure 4:**
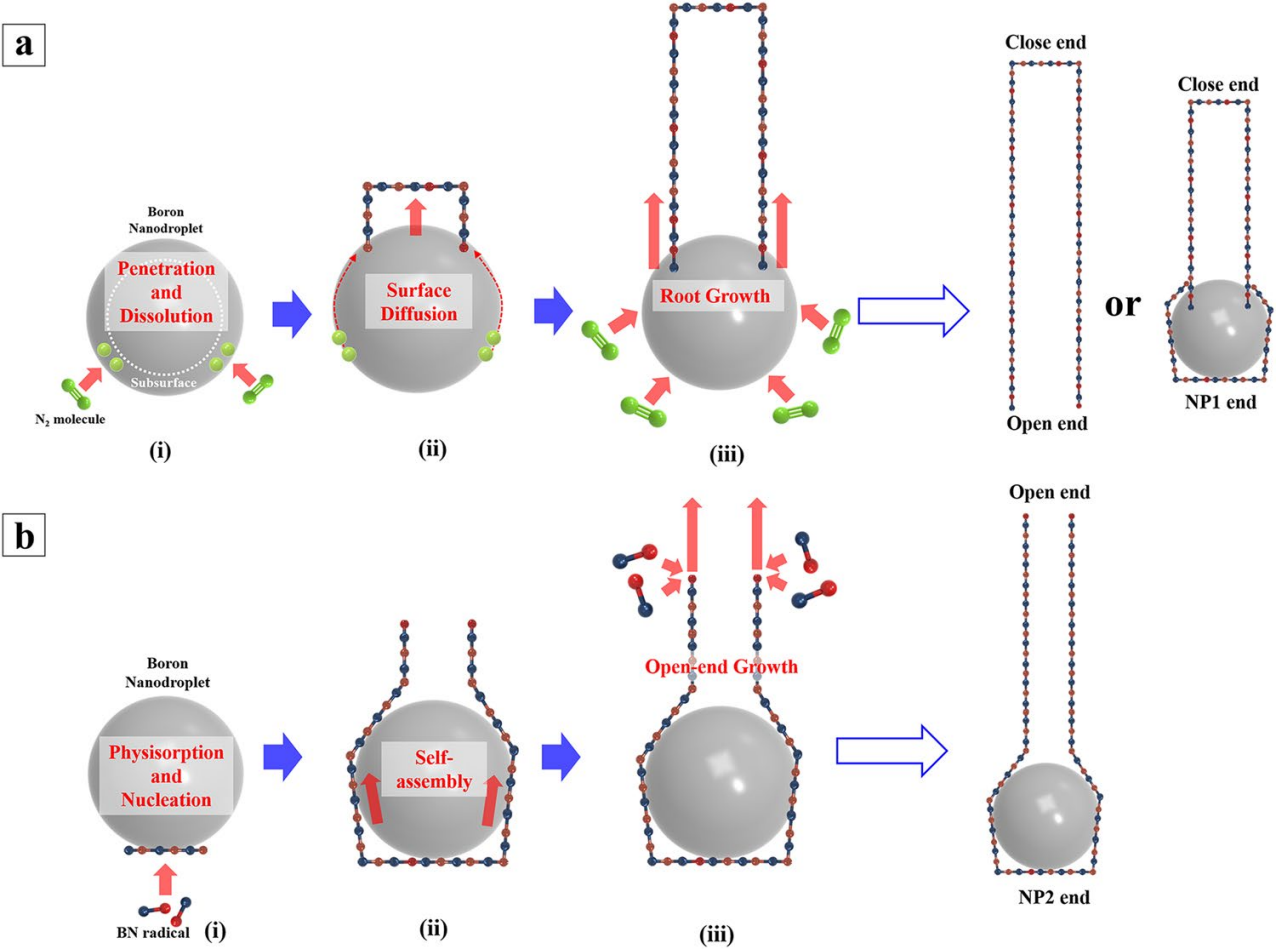
Schematic of (**a**) root growth and (**b**) open-end growth processes. (**a**) In root growth, a BN nanotube evolved from pre-formed BN cap on the surface of boron nanodroplets. Tube growth was sustained by the supply of nitrogen atoms at the root of the tubular structure. At the end of the process, BNNTs would have the NP1 end or open end if boron nanodroplets were completely consumed. (**b**) Open-end growth, on the other hand, started from polygonized h-BN layers formed from the nucleation of physisorbed BN radicals. Boron nanodroplets were kept isolated during the growth process, and the growth was maintained by the physisorption of BN radicals at the open end.

As shown in Fig. 4a, (i) N_2_ molecules from the surrounding penetrate into the subsurface of boron nanodroplets that are condensed from boron vapor that has evaporated from the surface of the macroscopic boron target. Because of the high chemical reactivity in the liquid phase^48–50^, boron can dissociate nitrogen molecules into individual atoms^47^. (ii) These nitrogen atoms migrate on the surface under the influence of surface diffusion, forming BN species, and subsequently assemble into a hexagonal structured BN cap. The nitrogen atoms on the outer surface of the boron droplet diffuse towards the boundary of the BN cap, where they combine with boron atoms, allowing the cap to progressively evolve into a cylindrical structure. (iii) A BNNT is subsequently elongated out of the nanodroplets and grows because of the continuous incorporation of nitrogen and boron atoms at the root of the tubular structure.

The NP 1 end structure of the BNNT in Fig. 3c can be explained by the root growth mechanism. In this process, the boron nanodroplets serve not only as a growth support but also as a boron reservoir; therefore, they will be steadily consumed when the temperature is in favor of tube growing, and if the growth happens long enough, these nanodroplets could be eventually devoured, leaving the end of BNNTs opened (Fig. 3b). Otherwise, h-BN layers can be formed from the nucleation of surface BN species when boron nanodroplets are solidified in a low temperature regime. The initial step of root growth involves interaction between nitrogen molecules and liquid boron droplets; however, there have not been many systematic studies of nitrogen behavior on the surface of boron droplets that directly leads to the formation of a boron nitride tubular structure. Recently, a theoretical model was developed by Santra *et al*. to simulate the root growth of BNNT in arc discharge^47^. In their study, the dynamics of nitrogen atoms and the formation of a tubular structure from a BN cap on the surface of boron droplets was demonstrated in a way that genuinely supported the root growth mechanism. The optimal temperature regime facilitating the tube’s growth ranged narrowly from 2000 to 2400 K, although their simulation was carried out in a sub-atmospheric pressure condition where the temperature was the only factor governing growth process. The atomistic phenomena in laser ablation at an especially elevated pressure (14 bar) would be more intense when taking into consideration the influence of the pressure factor. We presumed that the nitrogen molecules in this condition are allowed to penetrate and dissociate in the subsurface region of boron droplets. In consequence, this would lead to a significant boost in the growth rate of BNNT because of the increase of superficial nitrogen atoms. Similarly, the penetration of nitrogen molecules also took place in a macroscopic boron target, where it resulted in the formation of multilayer h-BN films coated on the surface of the target after synthesis (Fig. S4).

Although there is a possibility that the root growth mechanism can produce open-end nanotubes when boron droplets are fully consumed, that is not likely to happen at the bottom part of the gas plume, considering that the root growth will be disrupted when deposited on the low-height collector (1 cm). Also, it is worth mentioning that the NP2 end structure (Fig. 3d), in which the tubular structure grows from the outer h-BN layers and the growth of BNNTs seemed not to be affected by reactive liquid boron droplets, because the boron nanoparticle (NP2) was encapsulated inside the h-BN layers. Therefore, the root growth mechanism itself cannot fully explain the formation of a considerable proportion of the open and NP2 end structures of BNNTs in the high temperature region close to the boron macroscopic target (1 cm). The detailed proportion of BNNT ends will be discussed.

**Figure 5:**
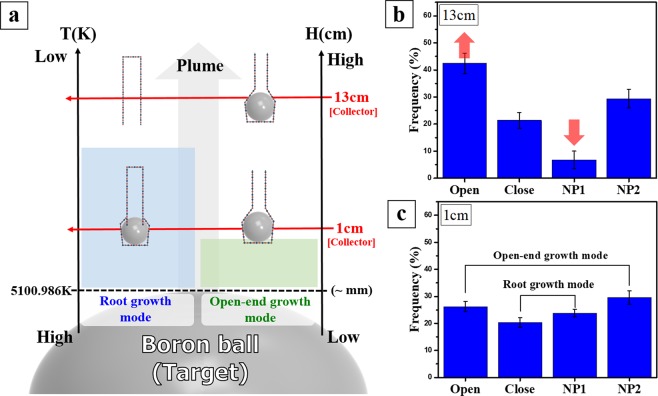
(**a**) Proposed growth regions for two growth modes in temperature and height profiles of the vertical gas plume. Both growth mechanisms were initiated when boron vapor condensed into liquid boron nanodroplets. Whereas the root growth can sustain higher positions, the open-end growth takes place around 1 cm above the macroscopic boron ball. The percentage of end structures in BNNT products collected at (**b**) top (13 cm) and (**c**) bottom (1 cm) parts of gas plume.

The second mechanism we proposed herein is based on heterogeneous nucleation and growth of BNNTs from the supersaturated flow of BN vapor, and is probably responsible for the formation of the majority of open and NP2 end BNNTs. To distinguish it from the root growth mechanism, we called it the “open-end growth” mechanism. The source of BNNT growth in this mechanism is the surrounding BN radicals originating from the gas-phase reaction B_2_ + N_2_ → 2BN (B_2_-N_2_ direct reaction) in the proximity of the macroscopic boron ball. Even in the extreme conditions in the laser ablation process, we suspect that a very high density of BN radicals would populate in the area very close to the boron ball. Gnoffo’s simulation model further theoretically confirms our speculation^38^. Referring to Fig. 5a, the simulation indicated that the high temperature a few millimeters above the boron ball combined with the high pressure environment could decompose molecular nitrogen and form a significant level of BN radicals at the base of the gas plume. These radicals decay into B_2_ and N_2_ when rising in a gas plume of mixed gases (B, B_2_, N, N_2_, BN), leading to the decrease of BN density in the higher area^38^. Similar to root growth, open-end growth starts when boron nanodroplets are condensed and advances in the following steps.

As shown in Fig. 4b, (i) the BN radicals are physically adsorbed on the surface of boron nanodroplets. When these radicals attain supersaturation, heterogeneous nucleation of h-BN layers on the outer surface will occur. (ii) Newly formed h-BN layers, grown by self-assembling of BN radicals and wrapping around boron nanodroplets, subsequently transform into the cylindrical structure extending from the NP2 end structure in Fig. 3d. (iii) BN radicals are continuously incorporated into the tubular structure at the growing open end. This is the physisorption of BN radicals on dangling bonds at the end of the nanotube.

The major difference between the open-end growth mechanism and root growth is the way BN species are supplied to the formation of the nanotube. Outer h-BN layers, once formed, will expansively cover boron nanodroplets, preventing them from interacting with molecular nitrogen in the open-end growth. For this reason, boron nanodroplets remain intact throughout a tube’s growth and simply serve as growth support. BN source for the growth process mainly comes from the surroundings, instead of from the boron droplet’s surface. Since the open-end growth mechanism exclusively relies on surrounding BN radicals, the significant depletion of BN density at a few centimeters above the macroscopic boron ball when boron nanodroplets move upward in the gas plume can disrupt the growth process, leading to the formation of the open-ended nanotube. To experimentally verify the proposed growth models, we collected BNNTs at two different parts of the vertical gas plume, 1 and 13 cm above the macroscopic boron target. As described above, once molten boron nanodroplet formed under 5101 K, the formation of BNNT will be initiated following root and open-end growth modes, simultaneously. However, most of BNNT growth by the open-end mode is finished at a low height, where BN radicals are populated with the highest density. On the other hand, the root-grown BNNTs will evolve continuously, as they rise up in the plume, as long as boron nanodroplets are maintained in a molten state, increasing the possibility that boron nanodroplets can be consumed entirely. The total process of dual growth modes in HTP laser ablation is shown in Fig. 5a. We compared BNNTs collected at 1 and 13 cm to identify the major difference between the two growth modes. In particular, the change in the end structure and the length of BNNTs will indicate the growth mode they followed.

The proportion of each type of end structures shows a noticeable difference between the bottom (1 cm) and top (13 cm) parts of the gas plume (See graph in Fig. 5b,c). In the BNNTs sample obtained at the bottom part, the NP ends are dominant with 53.4 ± 2.1%, followed up by 20.4 ± 1.8% of the closed end, and 26.2 ± 1.9% of the open end. In the NP-ended BNNTs population, NP 1 and NP 2 types contribute 23.8 ± 1.5%, and 29.6 ± 2.5%, respectively, whereas most of the BNNTs collected in the upper part (13 cm), up to 42.4 ± 3.8%, are open ended. The proportion of NP-ended nanotubes that made up 36.2 ± 4.1% of the total number of BNNTs includes 6.8 ± 3.2% of NP 1 and 29.4 ± 3.5% of NP 2 type. The percentage of closed-end BNNTs remains almost the same, with 21.4 ± 2.9% compared to that of the bottom-part sample. The statistical data for end structures of BNNT were acquired from direct observation of one hundred of nanotube ends in HR-TEM analysis. The data was acquired in five repeated measurements of each synthesized sample, and average data are presented.

To explain the differences of end structure between the BNNTs collected at the top and bottom part of the gas plume, the height profile of temperature should be taken into consideration. In the root growth mode, the prerequisite condition for the growth of nanotubes is the liquid phase of the boron nanoparticles needed to trigger the dissociation of nitrogen molecules. Referring to the temperature profile from simulation modelling of HTP laser ablation^38^, the temperature in favor of the liquid state of boron element can be maintained at a few centimeters above the boron macroscopic target depending on the power of the laser heating. For the sample acquired at the height of 1 cm, the root growth of BNNTs is likely to be abruptly stopped when they are deposited on the collector hanging in the gas plume. One can expect that in this case, boron nanodroplets, which will not be expended entirely because of the cease of the tube’s growth, are ultimately be attached at the end of the nanotube (NP 1 end) and the opposite side is closed end. Also, the highest density of BN radicals near the surface of the boron ball would strongly promote the second growth mode of BNNT. Therefore, the combination of disrupted root growth with unconsumed nanodroplets and the open-end growth mode leads to the predominance of BNNTs having a nanoparticle-containing end, which account for half of the total number of obtained BNNTs. As described above, the NP 1 end (23.8 ± 1.5%) and close-ended nanotube (20.4 ± 1.8%) are distinct features of root growth, whereas the NP 2 end (29.6 ± 2.5%) and open-ended nanotube (26.2 ± 1.9%) belong to open-end growth (Fig. 5c). From the calculated proportions for each kind of end structures, the total number of the open-end grown BNNTs is larger than that of the root-grown BNNTs, indicating the higher efficiency of the open-end growth mechanism compared to the other in the bottom region of the gas plume. It should be noted that, because of the exceptional length of BNNTs, we were unable to observe the whole nanotube with both ends in TEM analysis; therefore the proportions of end structures featured in a particular growth mechanism are not necessarily equal; i.e., NP 1 vs. close end in root growth, or NP 2 vs. open end in open-end growth. On the other hand, the proportions of BNNT end structures obtained at the top position in the gas plume (13 cm) changed noticeably. Although the proportions of NP 2 end and closed end remained unaffected, there was a conversion from NP 1 end to the open-end structure. The percentage of NP 1 end was reduced from 23.8 ± 1.5% to 6.8 ± 3.2%, corresponding to the equal amount of increase in the proportion of open-end nanotubes, which is from 26.2 ± 1.9% to 42.4 ± 3.8% (Fig. 5b). As discussed in the previous section, the open-end growth mode of BNNTs hardly occurs in the top part of the gas plume, thus, it does not contribute significantly to the specific differences in the end structures of BNNTs. The main reason for the conversion should come from the prolongation of the root growth mode. The root growth can reach its limit as the boron nanodroplets rise along the gas plume, wholly passing the liquid phase, increasing the possibility that boron nanodroplets can be consumed entirely. The depletion of the boron reservoir results in the absence of boron nanoparticle at the end of the nanotube, leaving the tube opened.

According to our proposed mechanism, root growth must involve the dissociation of nitrogen molecules in the subsurface of boron nanodroplets, and this kind of process typically requires high activation energy, which is dependent on temperature and pressure. This is fundamentally in contrast to the open-end growth mechanism, which does not require that much activation energy, because BN radicals incorporated in the growth process are physically absorbed from the surroundings. Thus, we expected that root growth might happen more slowly than open-end growth mode does. As a result, BNNTs originating from the former are shorter than are BNNTs following the latter. To confirm this hypothesis, we measured the length of separated BNNTs uniformly coated on a silicon substrate.

**Figure 6:**
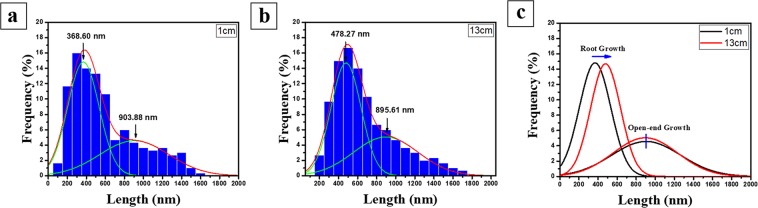
Length-distribution graph of obtained BNNTs (n = 500) at (**a**) 1 cm and (**b**) 13 cm in the vertical gas plume. The graph indicates that the length of BNNTs is distributed in two distinct regions, corresponding to the root growth and open-end growth mode. (**c**) The length-distribution curve in the root growth region shifted towards higher values for BNNT collected at 13 cm, but remained unchanged in open-end growth region.

In both samples, the lengths of BNNTs differed in a wide range, but were mainly distributed in two distinct regions, as shown in Fig. 6. As mentioned before, because of the difference in growth rate, the short BNNT domain in the length-distribution curve should be assigned to root growth, whereas the long BNNT domain is appointed to the open-end growth mode. In the root growth domain, the average length of BNNTs collected at the upper part increased by approximately 30%, compared to that of BNNT collected at the bottom part. The root growth mode will proceed as long as boron nanodroplets are maintained in the molten state. When we collected the sample at the height of 1 cm, the growth was generally terminated and unable to reach its limit, whease in the sample obtained at the higher position, the root growth was sustained until the boron nanodroplets were solidified or depleted, thus resulting in longer BNNTs. On the other hand, in the open-end growth domain, the average lengths of BNNTs collected at the top and bottom were almost comparable (903.88 nm, and 895.61 nm, respectively). As discussed above, this mechanism is the result of the adsorption of surrounding BN radicals onto boron nanodroplets. Because BN radicals were distributed predominantly in the proximity of the boron ball, we predicted that the open-end growth process may take place mainly in this area; so there would be no difference in the length of BNNTs acquired at the top and bottom parts of the gas plume. The spatial expansion of the BN radicals by additional plasma might possibly increase the yield and length of BNNTs, but as of now this remains as a future study.

## Conclusions

In summary, we investigated the growth mechanism of BNNTs in high temperature pressure (HTP) laser ablation. Two growth modes, the root growth mechanism, and the open-end growth mechanism were proposed based on the differences in end structures of synthesized BNNTs. In the root growth mechanism, BN radicals created from the dissociation of nitrogen molecules in the subsurface of condensed boron nanodroplets contribute substantially to the formation of BNNTs at the interface between the BN cap and the boron nanodroplets. On the other hand, the open-end growth mechanism relies on the physisorption of BN radicals arising from the gas-phase reaction between the boron vapor and nitrogen molecules in the proximity of the boron ball. BNNTs grown by this mechanism consistently start growing from h-BN layers that are coating boron nanodroplets. The difference in measured proportions of end structures of BNNTs collected at different positions in the vertical gas plume suggests that the open-end growth occurs only in the area close to the boron ball’s surface, approximately 1 cm above, whereas root growth can be sustained at higher positions. Furthermore, two distinct domains in the length distribution curves extracted from BNNT length measurements further confirm the coexistence of the two mechanisms in laser ablation. The short BNNT domain in length distribution is attributed to the root growth process, whereas the long BNNT domain is ascribed to the open-end growth process. It is understandable that, because the activation energy in the physisorption of BN species on the surface of boron droplets is not considerable, the rate of open-end growth is expected to be higher than that of root growth. This could be the clue to the production of long BNNTs by exploiting the open-end growth process. We are aware that BNNT synthesis in HTP laser vaporization may involve many complex processes at the nanoscale, and the two growth modes proposed in this study may not adequately reflect the formation of BNNTs in extreme conditions. Despite this fact, we expect that our understanding of the growth mechanism of BNNT in this study may pave the way for mass production of BNNTs in a controlled manner. Furthermore, the two types of BNNTs (open and closed end) discovered in this study would be specifically used corresponding to their structural characteristics. For example, open-ended BNNTs could be used as channels in filters for water treatment^51,52^ providing a suitable molecular path, whereas the close-ended BNNTs could be used as a field emitter^53^ to increase the stability applying the dangling-bond-free cab structures to prevent oxidation. We expect that the unique properties and structures of BNNTs can be exploited in substantially more fields with increasing productivity.

## Supplementary information

Supplementary information
