# Supplementary material for: Dual growth mode of boron nitride nanotubes in high temperature pressure laser ablation
Source: Sci Rep. 2019 Oct 30;9:15674. doi: 10.1038/s41598-019-52247-w (PMC6821736; doi:10.1038/s41598-019-52247-w)
Supplement: Supplementary file 1 — Supplementary information [file 41598_2019_52247_MOESM1_ESM.docx]

Supporting Information

Dual growth mode of boron nitride nanotubes in high temperature pressure laser ablation

*Jun Hee Kim^1,3^, Hyunjin Cho^1,6^, Thang Viet Pham^1,7^, Jae Hun Hwang^1,4^, Seokhoon Ahn^1^, Se Gyu Jang^1^, Hunsu Lee^2^, Cheol Park^5^, Cheol Sang Kim^3,4,*^ & Myung Jong Kim^1,7,*^*

^1^Functional Composite Materials Research Center, Korea Institute of Science and Technology, Wanju 55324, Republic of Korea.

^2^Composite Materials Applications Research Center, Korea Institute of Science and Technology, Wanju 55324, Republic of Korea.

^3^Department of Bionanosystem Engineering, Graduate School, Chonbuk National University, Jeonju 54896, Republic of Korea.

^4^Division of Mechanical Design Engineering, Chonbuk National University, Jeonju 54896, Republic of Korea.

^5^Advanced Materials and Processing Branch, NASA Langley Research Center, Hampton, Virginia, 23681, USA.

^6^Security and Disruptive Technologies Research Centre, National Research Council Canada, 1200 Montreal Road, Ottawa, Ontario, K1A 0R6, Canada.

^7^Division of Nano & Information Technology, KIST School, Korea University of Science and Technology, Seoul 02792, Republic of Korea

E-mail: [chskim@jbnu.ac.kr](mailto:chskim@jbnu.ac.kr) (C. S. Kim), [myung@kist.re.kr](mailto:myung@kist.re.kr) (M. J. Kim)

**Section 1. The measurement of molten boron ball by optical emission spectrometer (OES)**

By measuring the spectrum of thermal radiation from molten boron ball during the BNNT synthesis, we can calculate the temperature of boron target using Wien’s approximation.

$$I(\lambda,T)=2hc^{2}\cdot\lambda^{-5}\exp(-\frac{hc}{\lambda kT})$$

Where, *λ* is wave length, *h* is Plank’s constant, *c* is the speed of light and *K* is Boltzmann’s constant. *I(λ, T)* is the amount of energy per unit surface area per unit time per unit solid angle per unit wavelength emitted at a wavelength λ. The peak value of this curve occurs at a wavelength λ_max_ of

$$\lambda_{max}T=2.898\times{10}^{-3} mK$$

Therefore, the calculated temperature of boron ball during synthesis process is 3845.041K.

**Figure S1. Optical emission spectra of molten boron ball during laser ablation process at high pressure of 14 bar.**

**Section 2. Preparation procedure of BNNT length mesurement**


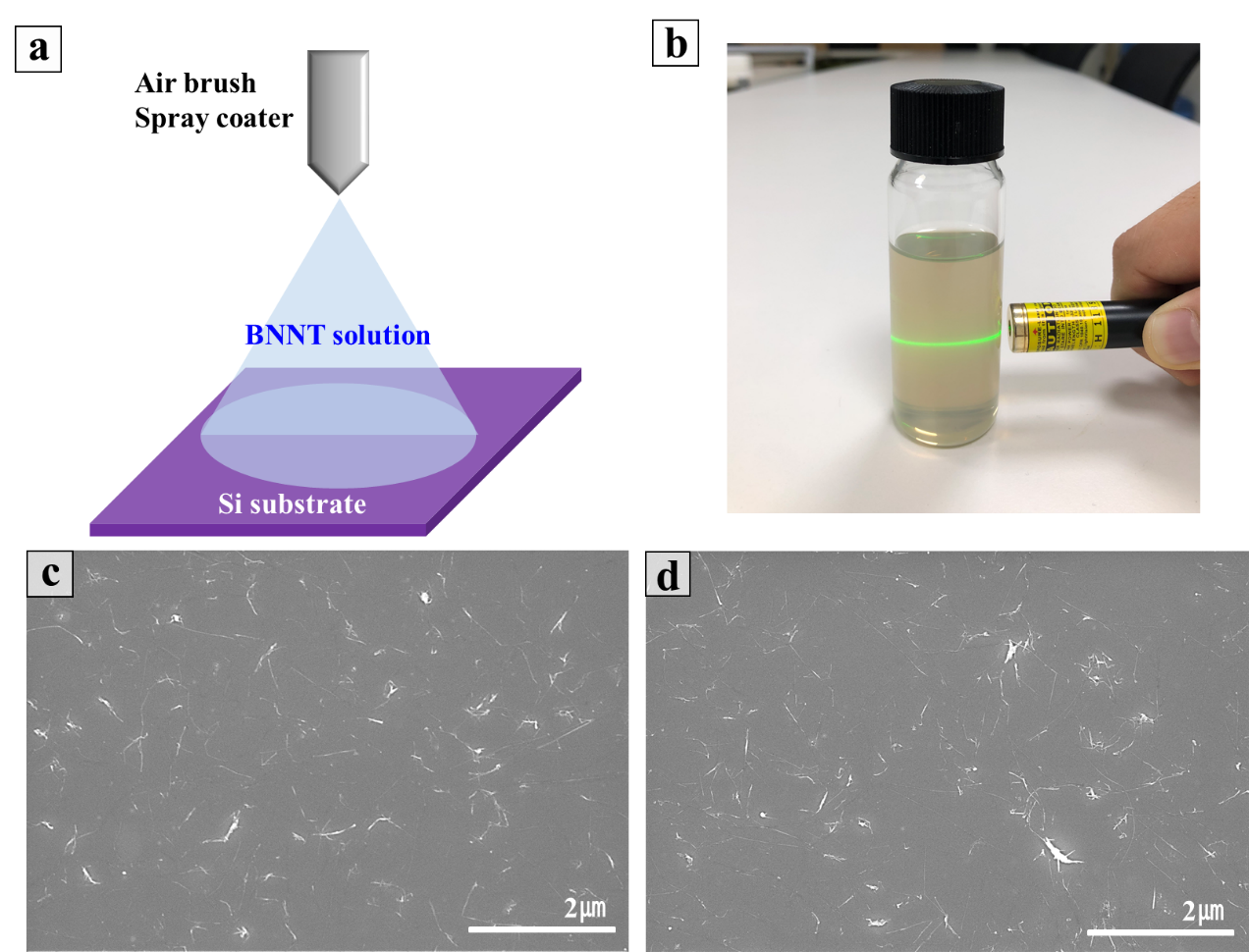


**Figure S2. (a) Schematic of preparation procedure of BNNT sample for length measurement using spray coating method. (b) Photo image of dispersed BNNTs in DMAc solvent. A laser pointer was irradiated through the solution, indicating good dispersion of BNNTs in DMAc solvent (0.25 mg/ml). FE-SEM images of spray coated BNNTs collected at (c) 1 and (d) 13 cm in vertical gas plume on a silicon substrate.**

**Section 3. The detailed microscope images of BNNTs**

**
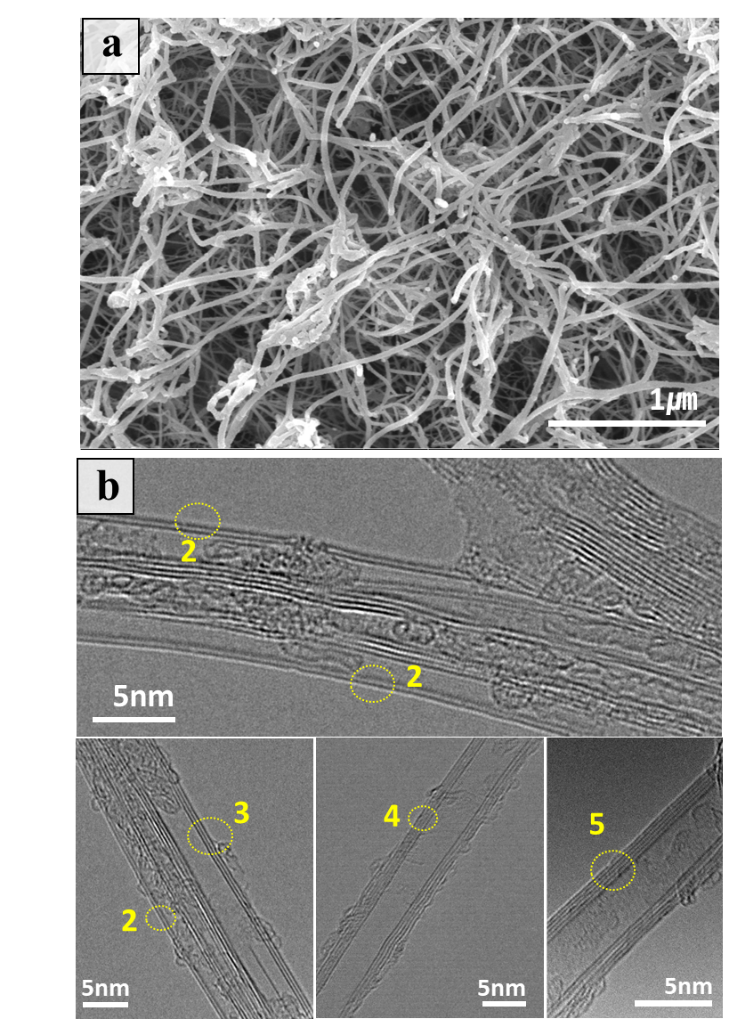
**

**Figure S3. (a) FE-SEM and (b) HR-TEM images of raw BNNTs showing the structure of BNNTs. (Yellow number is the number of wall layers)**

**Section 4. Surface analysis of macroscopic boron ball**

**
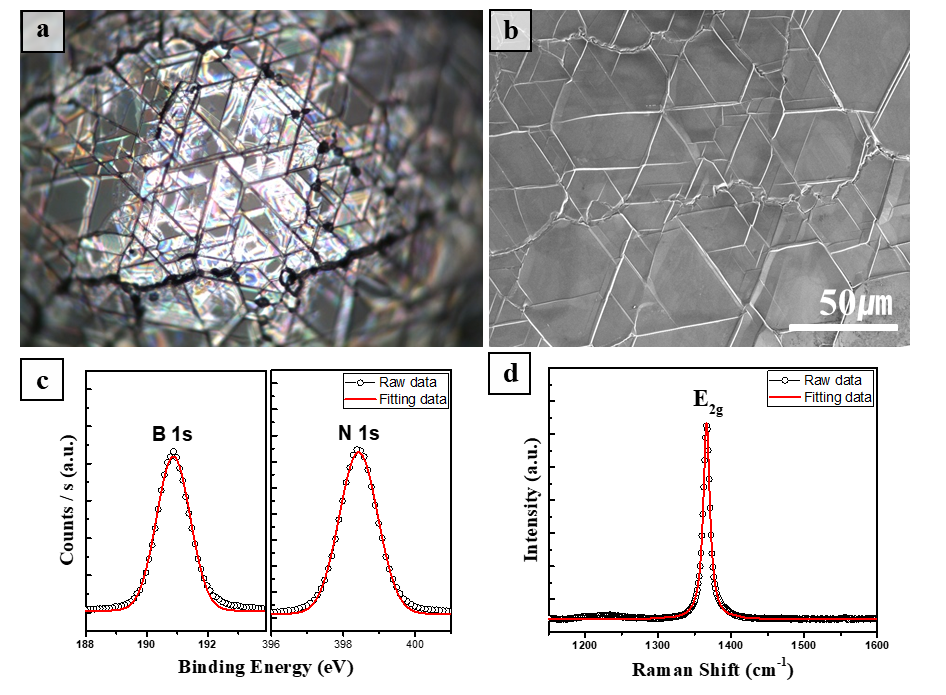
**

**Figure S4. (a) Optical image and (b) FE-SEM image of macroscopic boron ball. (c) High resolution B1s and N1s spectra of boron ball fitted with Gaussian curves,. (d) Raman spectrum of boron ball surface fitted with Lorentzian curve, showing E_2g_ phonon mode of hexagonal boron nitride structure.**

As shown in Fig. S3a and b, thesurface of macroscopic boron are covered by triangular domain layers. The bonding states and chemical composition of these layers were determined in XPS measurement (Fig. S3c). The stoichiometry B/N ratio was close to 1:1, and the binding energies of B1s and N1s spectra were 190.88 and 398.42 eV, respectively, which were in good agreement with reported values for h-BN^1^. Also, the Raman spectrum of the boron ball in Fig. S3d exhibited a single peak at 1365.9 cm^-1^ corresponding to E_2g_ phonon mode of a hexagonal boron nitride structure^2^. The formation of h-BN layers on the surface of macroscopic boron target is likely to be due to the recrystallization of surface BN molecules after laser ablation process.

REFERENCES

1 Shi, Y. *et al.* Synthesis of Few-Layer Hexagonal Boron Nitride Thin Film by Chemical Vapor Deposition. *Nano Letters* **10**, 4134-4139, doi:10.1021/nl1023707 (2010).

2 Gorbachev, R. V. *et al.* Hunting for Monolayer Boron Nitride: Optical and Raman Signatures. *Small* **7**, 465-468, doi:doi:10.1002/smll.201001628 (2011).
